# Resveratrol Attenuates Acute Inflammatory Injury in Experimental Subarachnoid Hemorrhage in Rats via Inhibition of TLR4 Pathway

**DOI:** 10.3390/ijms17081331

**Published:** 2016-08-12

**Authors:** Xiang-Sheng Zhang, Wei Li, Qi Wu, Ling-Yun Wu, Zhen-Nan Ye, Jing-Peng Liu, Zong Zhuang, Meng-Liang Zhou, Xin Zhang, Chun-Hua Hang

**Affiliations:** 1 Department of Neurosurgery, School of Medicine, Nanjing University, Jinling Hospital, 305 East Zhongshan Road, Nanjing 210002, China; zhangxssp@163.com (X.-S.Z.); lwxzlw@gmail.com (W.L.); njwuqi@gmail.com (Q.W.); dr.wulingyun@gmail.com (L.-Y.W.); zongzhuang@126.com (Z.Z.); mlzhou1979@hotmail.com (M.-L.Z.); 2 Department of Neurosurgery, School of Medicine, Southern Medical University (Guangzhou), Jinling Hospital, 305 East Zhongshan Road, Nanjing 210002, China; ringaly@126.com (Z.-N.Y.); 13677670746@163.com (J.-P.L.)

**Keywords:** toll-like receptor 4, subarachnoid hemorrhage, early brain injury, resveratrol, inflammation

## Abstract

Toll-like receptor 4 (TLR4) has been proven to play a critical role in neuroinflammation and to represent an important therapeutic target following subarachnoid hemorrhage (SAH). Resveratrol (RSV), a natural occurring polyphenolic compound, has a powerful anti-inflammatory property. However, the underlying molecular mechanisms of RSV in protecting against early brain injury (EBI) after SAH remain obscure. The purpose of this study was to investigate the effects of RSV on the TLR4-related inflammatory signaling pathway and EBI in rats after SAH. A prechiasmatic cistern SAH model was used in our experiment. The expressions of TLR4, high-mobility group box 1 (HMGB1), myeloid differentiation factor 88 (MyD88), and nuclear factor-κB (NF-κB) were evaluated by Western blot and immunohistochemistry. The expressions of Iba-1 and pro-inflammatory cytokines in brain cortex were determined by Western blot, immunofluorescence staining, or enzyme-linked immunosorbent assay. Neural apoptosis, brain edema, and neurological function were further evaluated to investigate the development of EBI. We found that post-SAH treatment with RSV could markedly inhibit the expressions of TLR4, HMGB1, MyD88, and NF-κB. Meanwhile, RSV significantly reduced microglia activation, as well as inflammatory cytokines leading to the amelioration of neural apoptosis, brain edema, and neurological behavior impairment at 24 h after SAH. However, RSV treatment failed to alleviate brain edema and neurological deficits at 72 h after SAH. These results indicated that RSV treatment could alleviate EBI after SAH, at least in part, via inhibition of TLR4-mediated inflammatory signaling pathway.

## 1. Introduction

Subarachnoid hemorrhage (SAH) is a fatal subtype of stroke with high mortality and morbidity rates. Early brain injury (EBI), which starts from the onset of SAH and lasts up to 72 h, is a major complication of SAH [1]. Accumulating evidence indicates that EBI is the main course of the poor outcome after SAH [2,3,4]. Therefore, novel treatment against EBI is believed to be a principal goal for patients with SAH [5,6]. It has been proven that a complex series of pathophysiological processes are involved in the pathogenesis of EBI. Among them, acute inflammatory injury plays an important role in EBI [4,7,8]. Therefore, prevention and reduction of inflammation may be a promising target for the treatment of SAH.

There are multiple signaling pathways that are activated early after the initial bleeding, which contribute to inflammatory response after SAH, are identified [7,8,9]. Among them, toll-like receptor 4 (TLR4), one of the most studied toll-like receptors (TLRs), plays an important role in initiating the inflammatory response following SAH [10,11]. Several stimuli can activate TLR4, including heat shock proteins, extracellular matrix degradation products, high-mobility group box 1 (HMGB1) [12]. Once activated, myeloid differentiation factor 88 (MyD88), a key adapter protein for TLR4, leads to direct activation of nuclear factor-κB (NF-κB) and the subsequent induction of prion-inflammatory cytokines implicated in the SAH-induced inflammatory responses [13]. In addition, activation of TLR4 could lead to cell death via NF-κB-dependent apoptosis [13]. Importantly, a growing number of experimental and clinical studies have demonstrated that TLR4 expression is upregulated in the brain. In addition, inhibiting TLR4 signaling pathway by pharmacological treatment can be against brain injury after SAH [14,15,16]. These indicate that suppressing TLR4 is a valid target for therapeutic intervention following SAH.

Resveratrol (RSV), a natural occurring polyphenolic compound, is widely distributed in grapevines, pines and legumes [17]. In recent years, its multiple functions including neuroprotective, anti-inflammatory, and anti-oxidant properties were extensively studied in different fields [18,19,20]. In vivo, RSV has been proven to cross the blood–brain barrier (BBB), and it has been proposed for the treatment of various neuroinflammatory and neurodegenerative diseases in the central nervous system (CNS) [21,22,23]. Thus far, although previous study reported that RSV could reduce neuroinflammation after SAH [22], the molecular mechanisms underlying RSV-dependent anti-inflammatory effects remain obscure. Accumulating studies indicated that RSV could inhibit the activation of TLR4 and the subsequent downstream signaling pathways in different fields [24,25,26]. Thus, we designed this study to confirm the hypothesis that RSV could attenuate SAH-induced EBI by modulating the TLR4 signaling pathway.

## 2. Results

### 2.1. Mortality Rates

A total of 156 rats were used in our experiment. Twelve rats died after the operation and were excluded from further analyses. No animals died in the sham-injured group and sham + RSV group. In the SAH + vehicle group, seven rats died and the mortality was 16.3%. Five rats died in the SAH + RSV group, and the mortality rate was 12.2%.

### 2.2. Effects of RSV on Neuroinflammation at 24 h Post SAH

**Figure 1:**
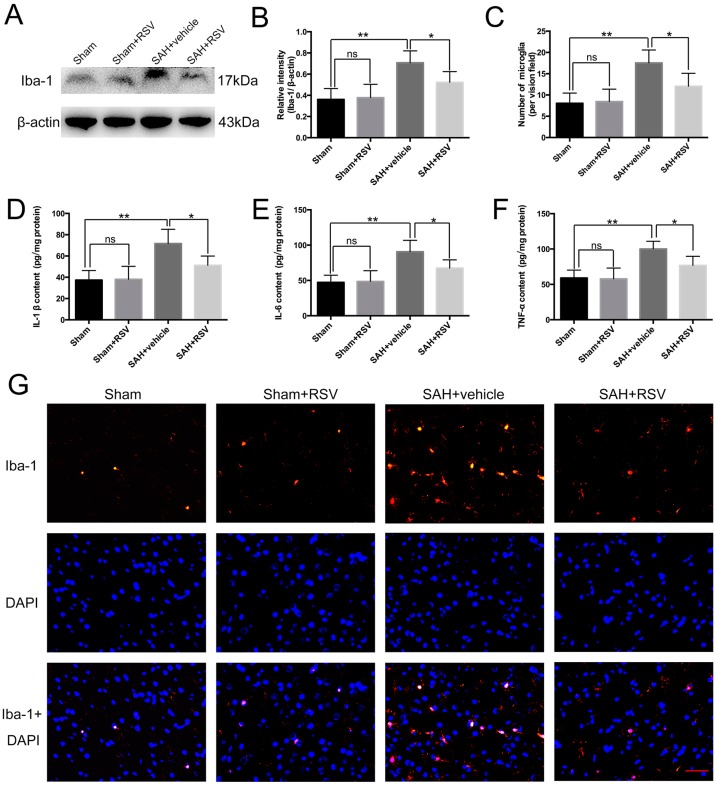
Effects of resveratrol (RSV) treatment on pro-inflammatory cytokine release and microglia activation at 24 h post subarachnoid hemorrhage (SAH). (**A**,**B**) Western blot analysis showing that RSV administration significantly suppressed Iba-1 (microglia marker) expression after SAH; (**C**,**G**) Immunofluorescence staining indicted that RSV could evidently reduce the number of activated microglia; (**D**–**F**) RSV markedly alleviated the expression of IL-1β, IL-6, and TNF-α in the brain cortex after SAH. Bars represent the mean ± SD. ** *p* < 0.01, * *p* < 0.05, and ns means non-significant. Scale Bars = 50 μm.

Activation of microglia is a critical source of pro-inflammatory cytokines in the brain. As shown in Figure 1A,B, there was a low level of Iba-1 expression in the sham group. SAH insults significantly increased the expression of Iba-1 when compared with sham group, but with RSV treatment, Iba-1 expression was markedly decreased. In addition, RSV markedly reduced the number of microglia in the rat cortex after SAH (Figure 1C,G). When the effects of RSV on the release of downstream inflammatory cytokines were evaluated, RSV administration after SAH was shown to significantly reduce IL-1β, IL-6, and TNF-α concentrations compared with SAH + vehicle group (Figure 1D–F).

### 2.3. Effects of RSV on the TLR4 Signaling Pathway

**Figure 2:**
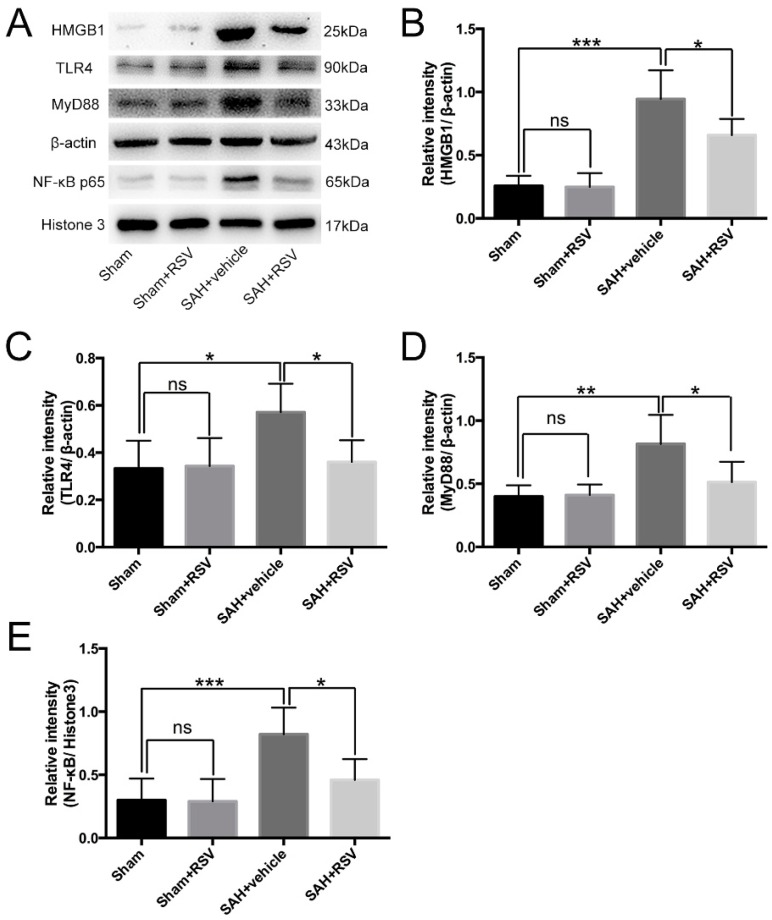
Effects of resveratrol (RSV) on the expression and activation of toll-like receptor 4 (TLR4)-related pathway 24 h post subarachnoid hemorrhage (SAH). (**A**) Representative Western blot images to detect the effects of RSV on the expressions of high-mobility group box 1 (HMGB1), TLR4, myeloid differentiation factor 88 (MyD88), and nuclear factor-κB (NF-κB); (**B**–**E**) Quantitative analyses of HMGB1, TLR4, MyD88, and NF-κB among all experimental groups. RSV treatment significantly reduced HMGB1, TLR4, MyD88, and NF-κB protein levels as compared with SAH + vehicle group. Bars represent the mean ± SD. *** *p* < 0.001, ** *p* < 0.01, * *p* < 0.05, and ns means non-significant.

**Figure 3:**
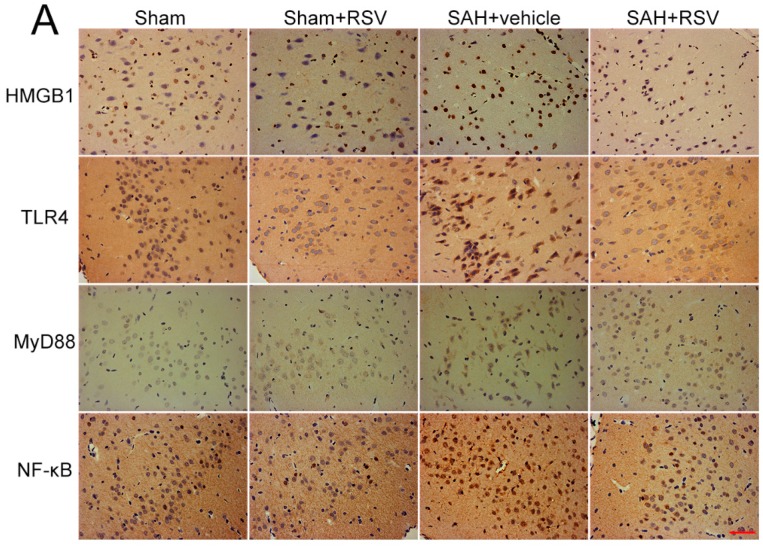

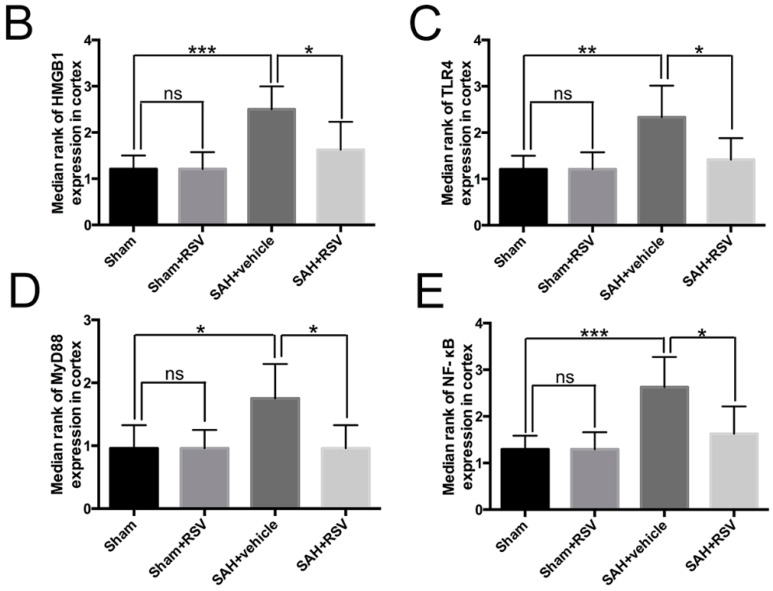
Effects of resveratrol (RSV) on high-mobility group box 1 (HMGB1), toll-like receptor 4 (TLR4), myeloid differentiation factor 88 (MyD88), and nuclear factor-κB (NF-κB) distribution at 24 h after subarachnoid hemorrhage (SAH). (**A**–**E**) RSV treatment could significantly reduce the immunoreactivity of HMGB1, TLR4, MyD88, and NF-κB in the cerebral cortex when compared with SAH + vehicle group. Bars represent the mean ± SD. *** *p* < 0.001, ** *p* < 0.01, * *p* < 0.05, and ns means non-significant. Scale Bars = 50 μm.

The expression and distribution of TLR4, MyD88, NF-κB, and HMGB1 were identified by Western blot analysis and immunohistochemistry. Results showed that SAH induced a marked increase in TLR4, MyD88, NF-κB, and HMGB1 expression in the brain samples, as compared with that in the sham group. Treatment with RSV significantly reduced the levels of TLR4, MyD88, NF-κB, and HMGB1 as compared with SAH + vehicle group (Figure 2A–E). To further confirm these results, brain sections were immunohistochemically stained with all the markers mentioned above. More TLR4, MyD88, NF-κB, and HMGB1 positive immunostained neural cells appeared in the SAH + vehicle group. After ATX administration, the immunoreactivity of TLR4-, MyD88-, NF-κB-, and HMGB1-positive cells was significantly decreased as compared with the SAH + vehicle group (Figure 3A–E).

### 2.4. Effects of RSV on Neural Apoptosis, Brain Edema, and Neurological Function at 24 h Post SAH

**Figure 4:**
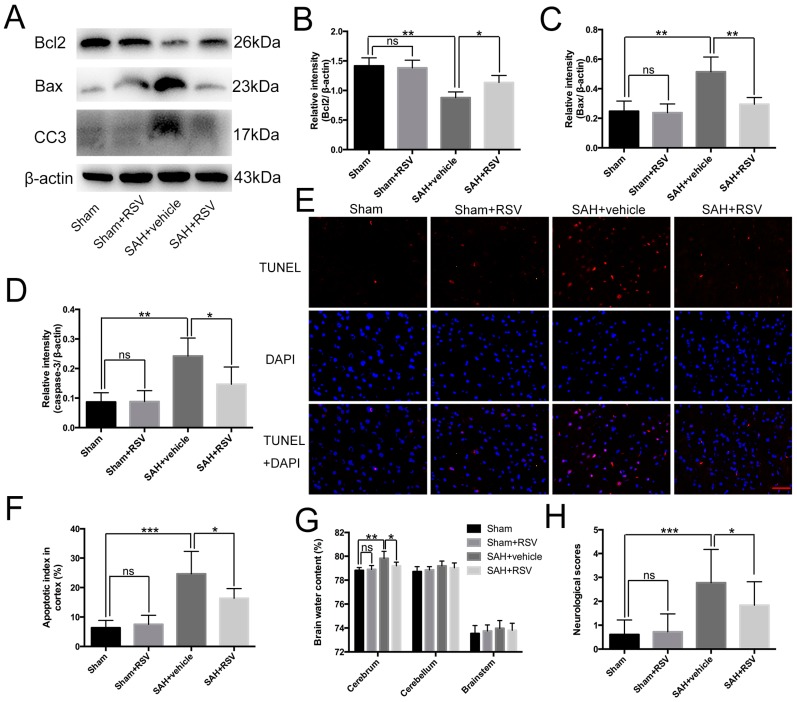
Effects of resveratrol (RSV) on neural apoptosis, brain edema, and neurological function at 24 h post subarachnoid hemorrhage (SAH). (**A**–**D**) RSV significantly reduced the elevated levels of cleaved caspase-3 and pro-apoptotic protein Bax, and enhanced the diminished level of Bcl2; (**E**,**F**) RSV administration markedly decreased the number of TUNEL-positive neural cells compared with the SAH + vehicle group; (**G**,**H**) RSV ameliorated brain edema and neurological behavior impairment at 24 h post SAH. Bars represent the mean ± SD. *** *p* < 0.001, ** *p* < 0.01, * *p* < 0.05, and ns means non-significant. Scale Bars = 50 μm.

Neural apoptosis and brain edema are two main components of EBI, which are responsible for poor outcomes after SAH [27]. As shown, at 24 h post-SAH, RSV evidently decreased the elevated levels of cleaved caspase-3 and Bax, and enhanced the diminished level of Bcl2 (Figure 4A–D). In addition, RSV administration after SAH significantly reduced the number of TUNEL-positive neural cells (Figure 4E,F). At the same time, RSV significantly reduced brain water content in the cerebrum as compared with the SAH + vehicle group (Figure 4G). For a better understanding of the efficacy of RSV for SAH-induced EBI, the neurological function was recorded. As expected, at 24 h post-SAH, the impairment of neurological function was significantly decreased when RSV was administered as treatment (Figure 4H).

### 2.5. Effects of RSV on Neural Survival, Brain Edema, and Neurological Function at 72 h Post SAH

**Figure 5:**
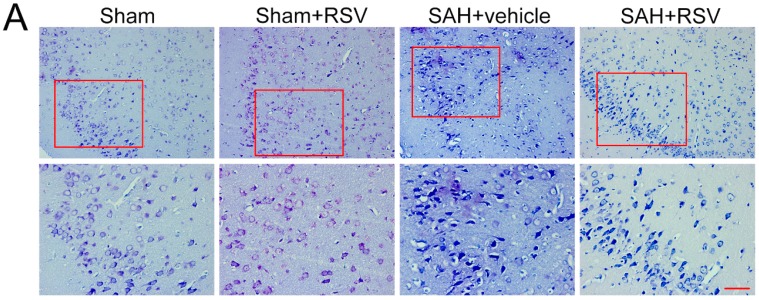

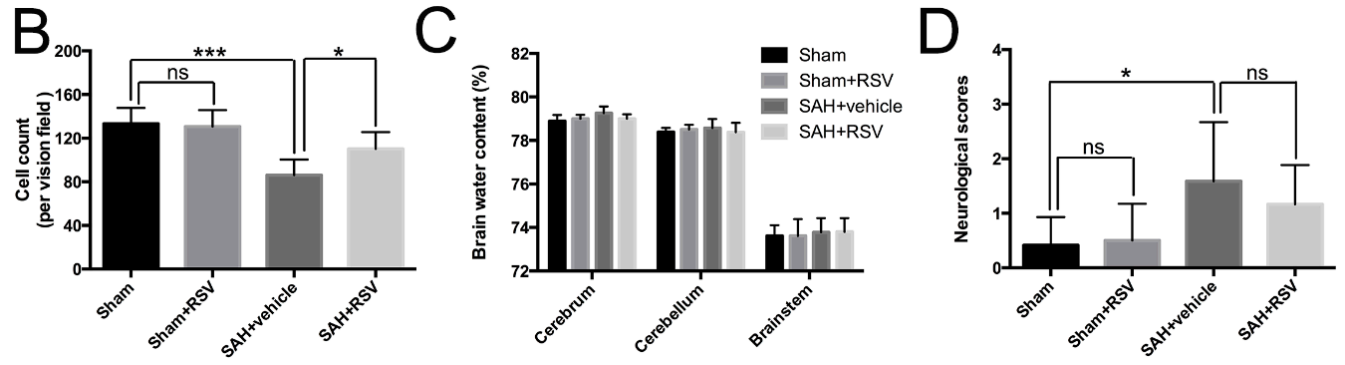
Effects of resveratrol (RSV) on neuronal survival, brain edema, and neurological function at 72 h post subarachnoid hemorrhage (SAH). (**A**,**B**) RSV treatment significantly increased the proportion of survived neurons compared with the SAH + vehicle group; Higher magnification of Nissl staining was shown in the red box for all groups; (**C**,**D**) RSV failed to alleviate brain edema and the impairment of neurological behavior compared with SAH + vehicle group at 72 h post SAH. Bars represent the mean ± SD. *** *p* < 0.001, * *p* < 0.05, and ns means non-significant. Scale Bars = 50 μm.

To further determine the possible long-term neuroprotective effects of RSV, neural survival, brain edema, and neurological function at 72 h after SAH were evaluated. As shown, considerable neuronal loss was observed in the SAH + vehicle group, which was significantly decreased when RSV was administered as treatment. Curiously, we found that there were no evident differences in brain edema and neurological function between SAH + vehicle group and SAH + RSV group (Figure 5C,D).

## 3. Discussion

In the present study, we studied the effects of RSV treatment on SAH-induced acute neuroinflammation and the possible role of the TLR4/MyD88/NF-κB pathway in the neuroprotective effects of RSV in SAH. The main findings can be summarized as follows: (1) RSV treatment ameliorated SAH-induced neuroinflammation, including microglia activation and pro-inflammatory cytokine release; (2) the TLR4/MyD88/NF-κB pathway was activated early after SAH, which could be inhibited by RSV treatment; and (3) after administration of RSV, SAH-induced neural apoptosis, brain edema, and neurological impairment were attenuated. These findings suggested for the first time that RSV could provide neuroprotection against SAH-induced neuroinflammation through inhibiting the TLR4/MyD88/NF-κB pathway.

Accumulating evidence indicates that neuroinflammation plays a key role in the pathogenesis of EBI, and that anti-inflammatory treatment may be beneficial in experimental or clinical SAH [4,7,8]. One key factor in the inflammatory response is microglia activation [14]. Microglia, the principle resident macrophages of the central nervous system (CNS), are involved in the innate immune response, producing and releasing a number of pro-inflammatory cytokines when activated [28]. These pro-inflammatory cytokines may not only cause direct damage to surrounding neural cells but also disrupt BBB permeability, exacerbate cerebral edema, and induce neural cell death to further exacerbate brain damage after SAH [6,28,29]. Consistent with the theory, we found marked increase of microglia activation and pro-inflammatory cytokine release leading to aggravated brain injury in the early period after SAH.

RSV is a natural polyphenol widely distributed in grapes and wine [30]. Recently, mounting evidence has demonstrated that RSV may serve as a promising therapeutic reagent in a variety of acute and chronic neurodegenerative diseases [17,20,31,32,33]. It has been proven that RSV has various pharmacological functions such as anti-inflammatory, anti-oxidant, anti-apoptotic, anti-bacterial, and anti-cancer properties [20,32,34,35,36]. Among these beneficial roles, the anti-inflammatory property is significant in the neuroprotective effect of RSV. In the brain injury models, including ischemic stroke, traumatic brain injury (TBI), and spinal cord injury (SCI), administration of RSV could remarkably reduce the levels of pro-inflammatory cytokines and microglia activation [32,33,37,38]. However, there is a paucity of studies exploring the anti-inflammatory effects of RSV in SAH. In the current study, we first evaluated the potential effects of RSV on neuroinflammation after SAH. We observed that RSV treatment could significantly inhibit the microglia activation and pro-inflammatory cytokine release in the brain cortex after SAH. Meanwhile, our data revealed that administration of RSV ameliorated neural apoptosis, brain edema, and neurological impairment at 24 h after SAH. These results strongly supported a neuroprotection role of RSV in SAH. However, the underlying mechanisms of RSV-dependent anti-inflammatory effects on EBI remain obscure.

TLRs, a family of pattern recognition receptors, play a pivotal role in the inflammatory response [13]. TLR4, a key member of the TLRs, is highly expressed on microglia [39]. It can be activated by endogenous ligands released from various cells, such as HMGB1 [12]. When activated, TLR4 initiates the MyD88-dependent pathway leading to direct NF-κB activation and induction of a number of pro-inflammatory genes and chemokines [13]. Most importantly, mountain evidence have demonstrated the critical role of the TLR4 signaling pathways on initiating an inflammatory response after SAH, and that inhibition of the TLR4 signaling pathways could attenuate microglia-induced neuroinflammation [14,15,30]. Regarding the relationship between RSV and TLR4, accumulating studies indicated that RSV could regulate the TLR4 pathway both in vivo and in vitro [24,26,38,39]. For example, Byun et al. (2015) reported that RSV could negatively regulate lipopolysaccharides (LPS)-induced NF-κB signaling through TLR4 in macrophages [26]. Li et al. (2015) demonstrated that RSV could attenuate inflammation in the rat heart subjected to ischemia-reperfusion by inhibition of the TLR4/NF-κB pathway [24]. Therefore, we hypothesized that RSV could protect against neuroinflammation induced by SAH through the inhibition of TLR4/MyD88/NF-κB signaling pathway.

In agreement with previous study [16], our data showed that the TLR4/MyD88/NF-κB pathway was activated and involved in the neuroinflammation in the early period after SAH. When treated with RSV, we found that the expression of TLR4-mediated agents, including HMGB1, MyD88, and NF-κB, was significantly inhibited. Given that these proteins play critical roles in SAH physiology [24,26,40,41], our experiment suggested that RSV could reduce neuroinflammation after SAH by inhibiting TLR4/MyD88/NF-κB signaling pathway. For a better understanding the neuroprotective effects of RSV after SAH, we further evaluated the neuroprotective effects of RSV at 72 h after SAH. Curiously, our data showed that RSV treatment could improve neural survival at 72 h after SAH. However, RSV treatment failed to ameliorate brain edema and neurological impairment at 72 h after SAH. We noted that there was no statistically significant difference in brain edema between sham and SAH groups. Thus, we speculated that the prechiasmatic SAH model used in our study might be not severity enough to discriminate statistical difference in different groups in the late period after SAH, although evident neural cell death could be seen at 72 h post SAH. The results suggested that more sever prechiasmatic SAH model or other SAH models might be needed to further evaluate the neuroprotective effects of RSV in the late phase of SAH.

Combining the research listed above, we speculated that RSV could regulate a complex series of inflammatory responses contributing to EBI after SAH through inhibiting the TLR4/MyD88/NF-κB signaling pathway. However, we cannot exclude other molecular mechanisms also involved in the neuroprotective effects of RSV in EBI. For instance, the nuclear factor erythroid-related factor 2 pathway, the sirtuin 1 pathway, and the mitogen-activated protein kinase pathway are all involved in the anti-inflammatory effect of RSV. Additionally, the therapeutic time windows of RSV after SAH remain obscure. Hence, future studies are warranted to address these issues.

## 4. Materials and Methods

### 4.1. Animal Preparation

Adult male Sprague-Dawley rats (250–300 g) were purchased from the Animal Center of Jinling Hospital (Nanjing, China). All procedures were approved by the Animal Care and Use Committee of Nanjing University (approval no. 2015013, June 2015) and were conformed to Guide for the Care and Use of Laboratory Animals by National Institutes of Health.

### 4.2. Prechiasmatic Cistern SAH Model

The prechiasmatic cistern SAH model was performed according to a previous study [42]. Briefly, the animal’s head was fixed in a stereotactic frame after intraperitoneal anesthetization with 10% chloral hydrate (0.35 mL/100 g). The hair on the head and near the inguinal region was shaved. After careful disinfection, a midline scalp incision was made and a 1 mm hole was drilled 8.0 mm anterior to the bregma in the midline. Approximately 0.35 mL non-heparinized fresh autologous arterial blood from the femoral artery was slowly injected into the prechiasmatic cistern in 20 s with a syringe pump under aseptic technique. Then, the burr hole was sealed with bone wax, and the incision was surgically sutured. The animals were then kept in a 30 °C heads-down position for 20 min, after which the rats were returned to their cages and housed at 25 °C for recovery from anesthesia.

### 4.3. Experimental Groups

**Figure 6:**
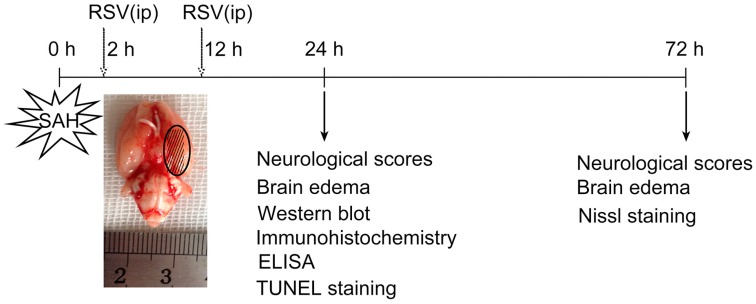
Schematic illustration of the experiment design. Resveratrol (RSV) was administered intraperitoneally (ip) at 2 h and 12 h after initial bleeding. In the first set of experiments, neurological scores, brain edema, Western blot analysis, immunohistochemistry, and TUNEL apoptosis were evaluated at 24 h after subarachnoid hemorrhage (SAH). In another set of experiments, neuronal survival, brain edema, and neurological function were evaluated at 72 h after SAH to determine the possible long-term benefits of RSV.

As shown in Figure 6, rats were randomly divided into four groups: a sham group (*n* = 36); a sham + RSV group (*n* = 36); a SAH + vehicle group (*n* = 36); and a SAH + RSV group (*n* = 36). In the animals of sham + RSV and SAH + RSV groups, RSV (Sigma-Aldrich, St. Louis, MO, USA) was formulated with 1% dimethylsulfoxide (DMSO) and physiological saline, and was given by intraperitoneal administration at a dose of 60 mg/kg at 2 and 12 h post-injury. The dose was selected according to our previous study [40]. Rats in SAH + vehicle group received an equal volume of vehicle, also by means of intraperitoneal injection at the same time points as mentioned above. Sham operation animals were injected with 0.35 mL saline instead of blood into prechiasmatic cistern.

In the first experiment setting, the animals were killed at 24 h after surgery. Post-assessments included neurological function, brain edema, enzyme-linked immunosorbent assays (ELISA), molecular tests, and histopathology. In the second experiment, a separate cohort of rats was used to further evaluate the possible long-term benefits of RSV. Neuronal survival, brain edema, and neurological function at 72 h after SAH were investigated.

### 4.4. Perfusion Fixation and Tissue Preparation

All rats were anesthetized and perfused through the left cardiac ventricle with 0.9% normal saline solutions (4 °C) until effluent from the right atrium was clear. The brain tissue was harvested on ice, and the temporal lobe tissue adjacent to the clotted blood was used for analysis. The brain samples were stored at −80 °C for Western blot and ELISA. For immunohistochemistry and immunofluorescence, rats were perfused with 0.9% normal saline (4 °C) followed by 4% buffered paraformaldehyde (4 °C), after which brains were  immersed in 4% buffered paraformaldehyde (4 °C).

### 4.5. Enzyme-Linked Immunosorbent Assay

The frozen brain samples were mechanically homogenized in 1 mL lysate buffer and centrifuged at 12,000× *g* for 20 min at 4 °C. The supernatant was then collected and total protein determined using a bicinchoninic acid assay kit (Bio-Rad Laboratories, Hercules, CA, USA). The inflammatory mediator’s protein levels were quantified using ELISA kits according to the manufacturer’s instructions (R&D Systems, Minneapolis, MN, USA) and cytokines concentrations within the brain tissue were expressed as picogram per milligram protein.

### 4.6. Total/Nuclear Protein Extraction and Western Blot Analysis

Rat brain tissue total/nuclear protein was extracted from rat brains using an established protocol from our laboratory [41]. The protein concentration was estimated by the method of Bradford with a standard commercial kit (Bio-Rad Laboratories, Hercules, CA, USA). For Western blot analysis, equal protein concentrations per lane were separated by 10% SDS-PAGE and transferred to polyvinylidene-difluoride (PVDF) membrane. After blocking, the membrane was incubated with primary antibodies against TLR4 (1:200, Santa Cruz Bio-Technology, Santa Cruz, CA, USA), MyD88 (1:200, Santa Cruz Bio-Technology, Santa Cruz, CA, USA), NF-κB P65 (1:200, Santa Cruz Bio-Technology, Santa Cruz, CA, USA), HMGB1 (1:1000, Cell signaling Technology, Beverly, MA, USA), Bcl-2 (1:200, Santa Cruz Bio-Technology, Santa Cruz, CA, USA), Bax (1:200, Santa Cruz Bio-Technology, Santa Cruz, CA, USA), caspase-3 (1:500, Cell Signaling Technology, Beverly, MA, USA), Histone-3 (1:3000, Bioworld Technology, Minneapolis, MN, USA), and β-actin (1:4000; Bioworld Technology, Minneapolis, MN, USA). The membranes were then incubated with goat anti-rabbit horseradish peroxidase (HRP)-conjugated IgG (1:5000). The blotted protein bands were visualized by using the enhanced chemiluminescence (ECL) Western blot detection reagents (Amersham, Arlington Heights, IL, USA). Quantification of band density was performed using the UN-Scan-It 6.1 software (Silk Scientific Inc., Orem, UT, USA).

### 4.7. Immunohistochemistry

For immunohistochemistry, brain sections (4 μm thickness) were incubated overnight at 4 °C with primary antibody against TLR4 (1:200), MyD88 (1:200), and NF-κB P65 (1:200) followed by a 15 min wash in phosphate buffered saline (PBS). After that the sections were incubated with HRP conjugated IgG (1:500) for 60 min at room temperature. Slides were visualized by incubated with 3,3′-diaminobenzidine (DAB) and hydrogen peroxide, followed by the assessment of staining intensity (five grades). “0” indicates that there were no detectable positive cells; “1” indicates very low density of positive cells; “2” indicates a moderate density of positive cells; “3” indicates a higher, but not maximal density of positive cells; and “4” indicates the highest density of positive cells.

### 4.8. Immunofluorescence Staining

Immunofluorescence was performed according to one of our previous studies [41]. Frozen tissue sections (6 μm thickness) were sliced and blocked with 5% normal fetal bovine serum in PBS containing 0.1% Triton X-100 for 2 h at room temperature, thereafter, sections were incubated with rabbit anti-Iba1 (1:100) overnight at 4 °C. Alexa Fluor 594 goat anti-rabbit IgG (1:200, Invitrogen, Shanghai, China) was used to detect the immunoreactivity of Iba1. 4-diamidino-2-phenylindole (DAPI) was used as a nuclear stain. Negative controls were prepared by omitting the primary antibodies. Fluorescence microscopy imaging was performed using ZEISS HB050 inverted microscope system and handled by Image-Pro Plus 6.0 software (Media Cybernetics, Rockville, MD, USA) and Adobe Photoshop CS5 (Adobe Systems, San Jose, CA, USA).

### 4.9. TUNEL Staining

Terminal deoxynucleotidyl transferase-mediated dUTP nick-end labeling (TUNEL) staining was performed according to the manufacturer’s instructions using an in situ cell death detection kit (Roche, South San Francisco, CA, USA). The number of apoptosis cells to DAPI was regarded as an apoptosis index (apoptosis cells/DAPI).

### 4.10. Nissl Staining

Nissl staining was performed to evaluate neuronal survival at 72 h after SAH. Brain sections (4 μm thickness) were hydrated in 1% toluidine blue for 10 min, washed with double distilled water, dehydrated and mounted with permount. Normal neurons have relatively big cell body, rich in cytoplasm, with one or two big round nuclei, whereas damaged cells have shrunken cell bodies, condensed nuclei, dark cytoplasm, and numerous empty vesicles.

### 4.11. Cell Counting

For each segment, six random high power fields (400×) in each coronary section were selected, and the mean percentage of positive cells in the six fields was used for final analysis. A total of four sections from each sample were used for quantification. All these processes were conducted by two investigators blinded to the experimental condition.

### 4.12. Neurological Scores

The clinical scores were recorded 24 and 72 h before being euthanized based on the independent observations by a veterinarian who was blinded to the experimental groups. Three behavioral activity examinations (Table 1) including appetite, activity, and neurological deficits were used in the scoring methodology.

### 4.13. Brain Water Content

Brain water content was measured at 24 and 72 h after surgery. Briefly, rats were anesthetized and decapitated, and the brains were quickly removed and separated into cerebrum, cerebellum and brain stem. Each part was immediately weighed as wet weight. Samples were then placed in an oven for 72 h at 100 °C before determining the dry weight. The percentage of brain water content (%) was calculated as follows: ((wet weight − dry weight)/wet weight) × 100%.

### 4.14. Statistical Analysis

All data were presented as mean ± SD. SPSS Statistics, version 19.0.0 (SPSS, Inc., Chicago, IL, USA) was used for statistical analysis of the data. All data were subjected to one-way analysis of variance (ANOVA) combined with Tukey post-hoc test. Statistical significance was inferred at *p* < 0.05.

## 5. Conclusions

In summary, we prove that RSV treatment exerts neuroprotection against SAH by combating with neuroinflammation, at least in part via inhibition of TLR4/MyD88/NF-κB-mediated signaling pathway. Our study suggests that RSV is an effective candidate for the treatment of SAH in a rat model.

## Figures and Tables

**Table 1:** Behavior scores.

Category	Behavior	Score
Appetite	Finished meal	0
	Left meal unfinished	1
	Scarcely ate	2
Activity	Walk and reach at least three corners of the cage	0
	Walk with some stimulation	1
	Almost always lying down	2
Deficits	No deficits	0
	Unstable walk	1
	Impossible to walk	2
